# Astroglial-axonal interactions during early stages of myelination in mixed cultures using *in vitro* and *ex vivo* imaging techniques

**DOI:** 10.1186/1471-2202-15-59

**Published:** 2014-05-02

**Authors:** Kalliopi Ioannidou, Kurt I Anderson, David Strachan, Julia M Edgar, Susan C Barnett

**Affiliations:** 1 Institute of Infection, Immunity and inflammation, College of Medical, Veterinary and Life Sciences, University of Glasgow, Sir Graeme Davies Building, 120 University Place, Glasgow G12 8TA, UK; 2 CRUK Beatson Institute, Garscube Estate, Glasgow G61 1BD, UK

**Keywords:** Astrocytes, Neurospheres, Time lapse, Glial/axonal interactions, Membrane blebs

## Abstract

**Backgound:**

Myelination is a very complex process that requires the cross talk between various neural cell types. Previously, using cytosolic or membrane associated GFP tagged neurospheres, we followed the interaction of oligodendrocytes with axons using time-lapse imaging *in vitro* and *ex vivo* and demonstrated dynamic changes in cell morphology. In this study we focus on GFP tagged astrocytes differentiated from neurospheres and their interactions with axons.

**Results:**

We show the close interaction of astrocyte processes with axons and with oligodendrocytes in mixed mouse spinal cord cultures with formation of membrane blebs as previously seen for oligodendrocytes in the same cultures. When GFP-tagged neurospheres were transplanted into the spinal cord of the dysmyelinated *shiverer* mouse, confirmation of dynamic changes in cell morphology was provided and a prevalence for astrocyte differentiation compared with oligodendroglial differentiation around the injection site. Furthermore, we were able to image GFP tagged neural cells *in vivo* after transplantation and the cells exhibited similar membrane changes as cells visualised *in vitro* and *ex vivo*.

**Conclusion:**

These data show that astrocytes exhibit dynamic cell process movement and changes in their membrane topography as they interact with axons and oligodendrocytes during the process of myelination, with the first demonstration of bleb formation in astrocytes.

## Background

Astrocytes (from the Greek word “astro” meaning star) are the most abundant cell type in the central nervous system (CNS) [1,2]. Astrocytes are important in various physiological processes in the CNS, such as synapse maintenance, blood brain barrier formation and after injury, in the formation of the glial scar [1,3-5]. Astrocytes are also important in facilitating CNS myelination [6-8]. The morphology of astrocytes is highly diverse, ranging from the extension of very few processes to many fine processes [9,10].

The discovery of neural stem cells in the adult mammalian brain [11] has facilitated the generation of large numbers of oligodendrocytes and astrocytes in culture and also has led to their therapeutic application in CNS diseases [12]. Stem and progenitor cells from a wide range of sources, including murine, primate and human fetal or adult tissues have been used for myelination and remyelination studies [13-16]. Mouse striatum-derived neurospheres have been used to study myelination after transplantation into *shiverer* mice for the study of glial-axonal interactions that culminate in myelin formation in the CNS [17-19]. Previously, we used advanced imaging techniques together with a range of green fluorescent protein (GFP) tagged glia and axons in transgenic mice to follow myelination *in vitro* and *ex vivo,* over time. These studies allowed the visualisation of oligodendroglial cell process movement during myelination and identified active membrane activity with the formation of blebs as myelination occurred [19]. Blebs are cellular protrusions that appear to aid in forward movement of the cells and their migration. They are believed to be instigated by hydrostatic pressure and depend on cellular mechanical properties and appear as spherical expansions of the membrane [20,21].

The differentiation and maturation of oligodendrocyte precursor cells (OPCs) is a prerequisite for CNS myelination during development and for remyelination in demyelinating diseases, while the underlying molecular mechanisms remain largely unknown [22]. Many studies have demonstrated that astrocytes promote CNS myelination in various culture models by secreting promyelinating factors [6,23,24]. Thus, it is of interest to examine how astrocytes in complex neural environments interact with neighbouring neural cells. Here we have taken three approaches in order to observe astrocyte interactions; the first is to use a complex myelinating murine cell culture containing axons and glial cells; the second is after transplantation in an *ex vivo* 3D environment and lastly after transplantation *in vivo*.

We find astrocytes are highly dynamic when cultured with axons and oligodendrocytes and can be seen to exhibit morphological changes compatible with bleb formation. Moreover, we have found that the transplantation of neurospheres into *shiverer* mice leads to a propensity for astrocyte differentiation near the injection site with more oligodendrocyte differentiation at distances further away. Lastly, we show similar highly dynamic changes in astrocyte-like cells in *ex vivo* and *in vivo* spinal cord.

## Results

### Differentiation of striatum-derived neurospheres to generate glial cells

**Figure 1:**
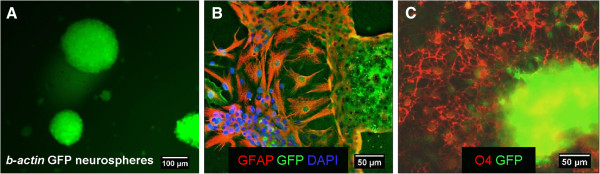
**Neurospheres differentiated into glial cells when cultured in differentiation medium *in vitro*. A)** cGFP-positive neurospheres isolated from *β-actin* GFP-transgenic mice expressing cytoplasmic GFP (cGFP), maintained 12 days in neurosphere medium *in vitro* were observed as free-floating aggregates consisting various sized spheres. Most cells within the sphere expressed cGFP. Upon withdrawal of growth factors and culture in differentiation medium for the myelinating culture, on PLL-coated coverslips for 3 days, the majority of neurospheres acquired antigenic properties of astrocytes, as shown with antibody to GFAP (red) **(B)** or oligodendrocytes as illustrated with the oligodendroglial marker O4 (red) **(C)**. In **B** and **C**, the endogenous GFP signal is enhanced with an antibody to GFP. Representative images from at least 3 separate experiments.

The multipotentiality of striatum-derived GFP-tagged neurospheres in differentiation medium was confirmed by determining their capacity to express astroglial and oligodendroglial markers *in vitro*. Striatum-derived neurospheres were generated as free-floating aggregates consisting of spheres of various sizes (Figure 1A). cGFP-positive neurospheres were harvested from the same flask and used for an *in vivo* transplantation or cultured on PLL-coated coverslips in the medium used to prepare the myelinating cultures. The GFP expressing neurospheres differentiated into GFAP positive astrocytes and O4 positive oligodendrocytes (Figure 1B,C) confirming the utility of these GFP expressing cells for the study of these cell types.

### Time-lapse imaging of neurosphere-derived labelled cell in myelinating cultures on 12 DIV (days *in vitro*)

**Figure 2:**
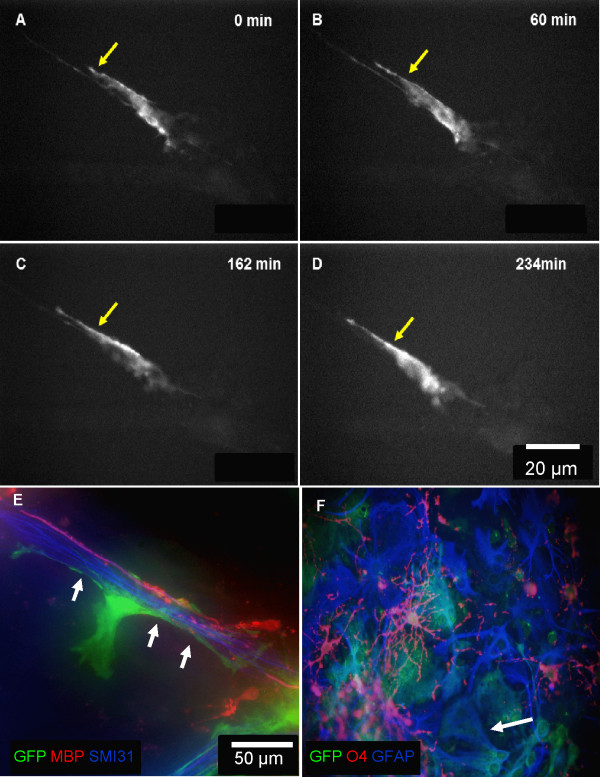
**Time-lapse imaging of elongation of a fGFP tagged astrocyte-like cell process.** fGFP infected neurospheres were added to myelinating cultures prepared from wild type embryos, on 12 DIV. Frames were captured from a 17 hours time-lapse sequence after 19 DIV. **A-D)** Times shown in upper right corner, represent time elapsed after the start of imaging. The culture was visualised every 3 min. A fGFP-positive cell was detected making contact with a bundle of neurites with continual movement along these neurite bundles as followed over time. The yellow arrow indicates the process extension which was variable, ranging from <1 μm to >15 μm. The process length changed during the first 160-min of the imaging sequence. **E)** Immunostaining of a sister myelinating culture for MBP (red) and for neurites (SMI-31, blue) showed the presence of myelin-like membrane in association with a GFP-positive astrocyte-like cell (green). **F)** Immunostaining of a sister myelinating culture for O4 (red) and for GFAP (blue) showed the presence of typical oligodendrocytes and flat astrocytes in association with a GFP-positive staining (green). Representative images from at least 4 separate experiments.

fGFP tagged neurospheres were added to myelinating cultures derived from wild type mice. The initial stages of interaction of the fGFP neurosphere-derived cells with the axonal surface were followed using time-lapse microscopy. Many labelled cells could be seen with a leading process projecting towards the axonal fascicle. Astrocyte-like cells had a leading process which was highly dynamic, extending and retracting slowly during the time studied. The leading process (arrow, Figure 2A-D) constantly sent out growth cone-like structures. Immunostaining of sister myelinating cultures showed GFP-positive, astrocyte-like cells closely associated with axons at an early stage of their myelination by oligodendrocytes in culture, as illustrated by the presence of MBP (Figure 2E) and GFAP and GFP positive astrocytes showing their typical flat morphology (Figure 2F).

### *In vitro* time-lapse observations of membrane changes of exogenous fGFP positive cells in wild type derived myelinating cultures

**Figure 3:**
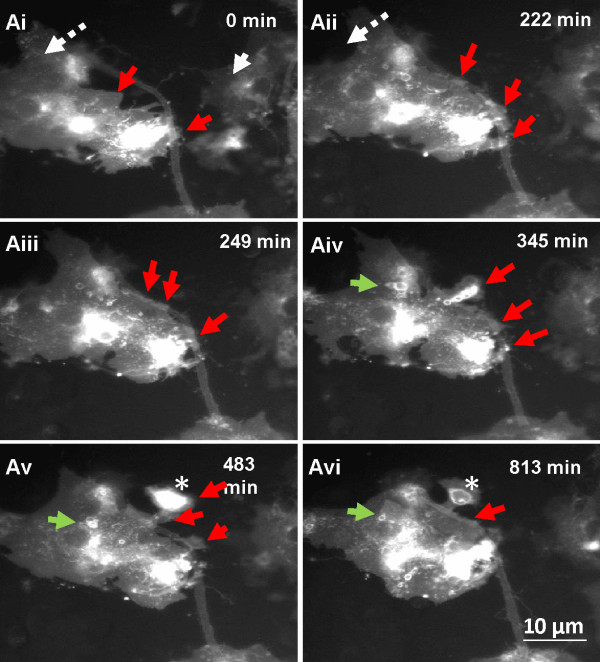
**Time-lapse imaging of membrane extension.** fGFP neurospheres were added in *shiverer* myelinating cultures and time-lapse imaging was performed on 22 DIV for 21 hours with 3 min time intervals. Ai -vi) Time-lapse sequence of a fGFP-positive cell moving dynamically like a wave towards a thin fGFP-positive *shiverer* axon (red arrows). During the recordings, numerous intracellular, round structures (green arrows) were imaged displaying a dynamic activity of the membrane of the fGFP-positive astrocyte-like cells. See Additional file 1, from 2 separate experiments.

Time-lapse sequence over 21 hours of a dynamic fGFP labelled process from flat astrocyte like cells in association with a neurite/axon-like structure. Over time, the central part of the membrane from a flat GFP labelled cell was seen to move towards a putative neurite eventually reaching the neurite surface at several points (red arrows, Figure 3Ai-ii). At a later stage of the time-lapse sequence the membrane seemed to contact the neurite (Figure 3Aii). Around the same time, a membranous protrusion of about 10 μm diameter formed from the main membrane which crossed over on the top surface of the neurite (Figure 3Aiv-v, arrow). Moreover, during the time-lapse imaging, numerous rounded GFP tagged protrusions or membranous blebs (asterisk), [20,21] were detected suggestive of an active motile, membrane (green arrows, Figure 3Civ-vi, Additional file 1).

**Figure 4:**
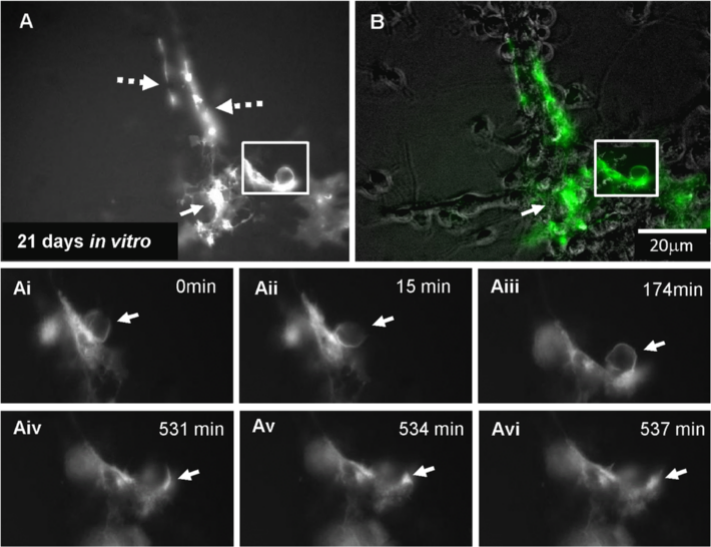
**Time-lapse imaging of membrane protrusions of fGFP-positive cells in *shiverer *cultures.** Time-lapse imaging was performed on 21 DIV for a period of 24 hours with 3 min time-interval. **A and B)** fGFP-labelled neurospheres appeared to have been differentiated into astrocyte-like cells (arrow) with processes aligned with the *shiverer* axons (broken arrows). Ai-vi) Time-lapse sequence of the magnified area (box) revealed the generation of membrane protrusions (arrows) which changed shape and appeared to be eventually deposited on the axonal surface. Additional files 2, 3 separate experiments.

Time-lapse images were obtained from another culture in which fGFP labelled neurospheres were added to *shiverer* cultures after 21 DIV. In Figure 4 membrane protrusion/blebs were seen in cells with morphology typical of astrocytes, (arrow, Figure 4A and B, bright field). These membrane protrusions were imaged in close contact with neurites. The fGFP-positive processes aligned over the neurites (broken arrows, Figure 4A). Membrane changes detected during the time lapse sequence included spherical protrusions/blebs that traversed the neurite surface, changing morphology and size (arrows, Figure 4Ai-vi, Additional file 2).

### cGFP neurospheres survive, integrate and differentiate into glial cells in the white matter of the *shiverer* mouse spinal cord

**Figure 5:**
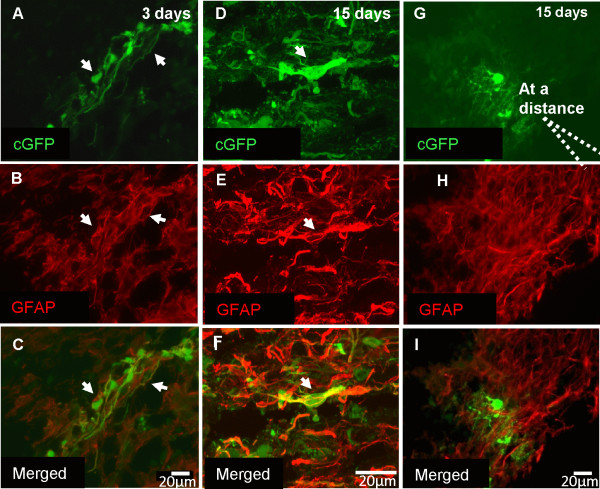
**cGFP-positive transplanted cells near the transplantation site differentiated into astrocytes.** Images were obtained from spinal cord of a *shiverer* mouse transplanted with cGFP positive neurospheres. **A-C)** 3 days post-transplantation of cGFP positive neurospheres many transplanted cells had differentiated into astrocytes, as indicated by colocalisation with the antibody to GFAP (arrow). **D-F)**. High magnification confocal images, revealed the morphological details of the cGFP-positive astroglial cells 15 days post-transplantation. **G-I)** A few microns from the transplantation point, the transplanted cells had morphology resembling that of oligodendrocytes with many fine processes. These cells were negative for GFAP. Representative images from at least 15 separate experiments.

To confirm the identity of cells in transplanted cords and to correlate cell morphology with cell type, cords were immunostained with cell type specific markers to identify the transplanted cells at 3, and 15 days post-transplantation. At both time-points many neurosphere-derived cells had differentiated into astrocytes at the injection site, as identified by immunostaining using an antibody to GFAP (Figure 5). The central area close to the injection site appeared to have a higher density of GFAP-positive cells than the perimeter (Figure 5D-I). A few microns from the transplantation point, transplanted cells that were negative for GFAP, exhibited a morphology resembling that of oligodendrocytes with many fine processes (Figure 5G-I).

### Distribution of the cGFP transplanted neurospheres into the spinal tissue of *shiverer* mouse

**Figure 6:**
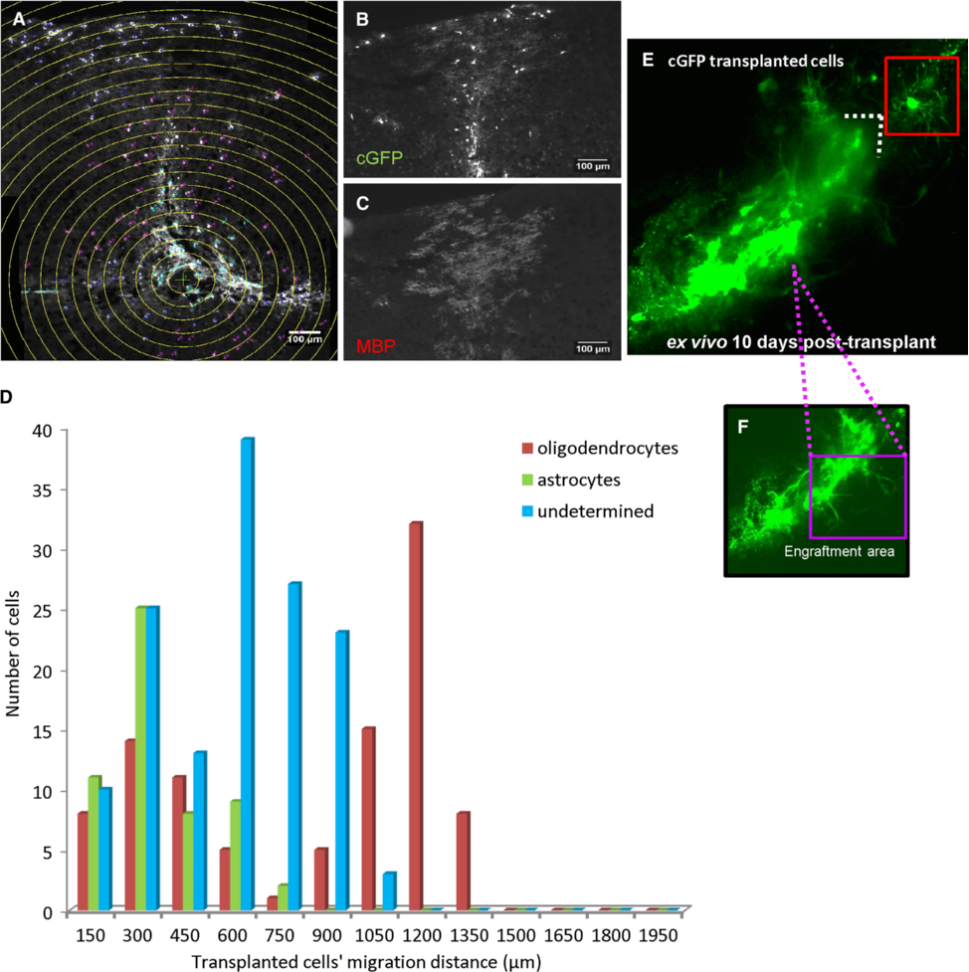
**Topographical study of cGFP-expressing cells 15 days post-transplantation. A)** Using Image J and a macro that created homocentric circles from the injection site (epicentre), the distance of the various cell types was measured in relation to the epicentre. **B-C)** A number of cGFP-positive transplanted cells, which colocalised with the MBP marker were found at a distance of 600 μm from the needle track. **D)** Graph shows the percentage of all cGFP-positive cells constituting oligodendrocytes, astrocytes or cells which were not positive for either the GFAP or MBP marker, according to their relative location from the needle track (n = 2). **E-F)** Tile scan using multiphoton microscopy of the transplanted area showed a dense cellular network comprised of cells having long processes (purple box, **F**) at the centre and the periphery of the injection site, typical of astrocytes. At a distance and at the bottom of the engraftment area, individual cells (Insert, red box) with clear symmetric, fine processes were detected resembling oligodendrocytes.

Cells were transplanted into the dorsal columns of the *shiverer* spinal cord, and were usually found at later stages extending rostro-caudally through these columns, largely though not exclusively in the ‘white’ matter (Figure 6A,B). Using Image J and a macro (generated in the lab) that created homocentric circles allocated from the epicentre of the injection area, the distance of oligodendroglial cells (MBP), astrocytes (GFAP) and other cells (GFAP/MBP–ve) from the transplantation site was measured. The transplanted cells were found at a distance of up to 600 μm from the injection site (Figure 6A). In many cases cGFP-positive cells (anti-GFP) were double labelled with anti-MBP and extended myelin-like sheaths. These cells were found both close to and at a distance from the needle track (Figure 6B,C). Close to the injection site, a similar proportion of transplanted cells had differentiated into GFAP-positive cells, while a minority of cGFP-positive cells could be detected that did not express GFAP or MBP. At a distance from the injection site, the majority of transplanted cells were positive for MBP and thin myelin-like sheaths were observed (Figure 6C). Quantification can be seen in Figure 6D.

*Ex vivo* imaging was performed of the transplant site and a tile scan of the whole area of revealed many cGFP-expressing cells bearing long processes forming a dense network at the centre and the periphery of the injection site (Figure 6E). The long tails (purple box) and the morphology of the processes of the cGFP-transplanted cells across the spinal tissue and especially at the engraftment area appeared typical of astrocytes (Figure 6F). At a distance from the main transplantation area, individual cells with many processes typical of oligodendrocytes were observed at the bottom of the engraftment region (insert, red box, Figure 6E).

### *Ex vivo* time-lapse of cGFP-expressing cells typical of astrocytes revealed their dynamic behaviour

**Figure 7:**
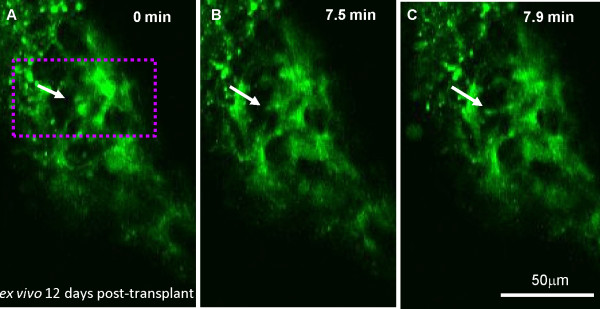
**Spontaneous transient extension of astroglial-like process *ex vivo*. A-C) ***Ex vivo* time-lapse of a non fixed spinal cord 12 days post-transplantation illustrates a process extending from a cGFP labelled astrocyte-like cell around the graft, over a period of less than 8 min. See Additional file 3. Representative videos from at least 2 separate experiments.

Visualisation of cGFP-expressing cells *ex vivo* in the spinal cord transplanted 12 days previously demonstrated that the processes of the exogenous cells were motile. Although, clarification of the identity of the visualised cells was difficult, previous staining of transplanted cord suggest the morphology is typical of astrocytes (Figure 5). Additionally, cGFP-expressing cells bearing fine processes typical of oligodendrocytes were usually found as single cells or in association with another one of similar morphology, in the plane of view [19]. On the contrary, cGFP transplanted cells with thick processes appeared to be part of a dense cellular network typical of astrocytes. Time-lapse imaging *ex vivo* over a short period of time revealed changes in the pattern of cGFP localisation suggesting a spontaneous extension of an astroglial-like processes (Figure 7A-C, Additional file 3).

### Live *in vivo* imaging of the dynamics of cGFP-transplanted cells after transplantation into the spinal cord of *shiverer* mice

**Figure 8:**
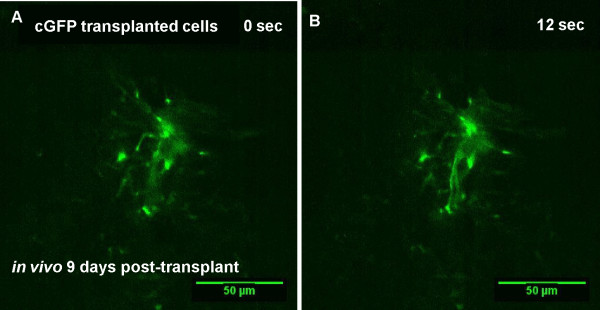
***In vivo *imaging of cGFP-labelled cells 9 days post-transplantation.***In vivo* time-lapse nine days post-transplantation, showed cGFP-labelled cells with long processes that resembled astrocytes. **A** and **B** shows change in fluorescence over time. See Additional file 4.

Our ultimate aim was to visualise transplanted neural cells in the spinal cord *in vivo.* Although we demonstrated this was possible, problems were associated with image acquisition due to movement caused by breathing in real time (Additional file 4). However, the mice survived well even after 5 hours of imaging. Figure 8 shows *in vivo* time-lapse imaging stills nine days post-transplantation, of cGFP-labelled cells with long processes (Figure 8A,B; Additional file 4).

## Discussion

We have carried out studies *in vitro*, *ex vivo* and *in vivo* to follow the interaction of astrocytes differentiated from striata derived neurospheres in either myelinating cultures or after transplantation into myelin deficient mice. As seen for oligodendrocytes, astrocytes have very dynamic membranes *in vitro, ex vivo* and *in vivo.* After transplantation of neurospheres into spinal cord dorsal columns, prevalence for astrocyte differentiation compared with oligodendroglial differentiation was seen around the injection site. These data show that astrocytes have dynamic membranes with active bleb formation associated with cell process movement and suggest this process may be important in their motility and cellular interactions.

To study the function of individual glial cells that are embedded in a complex neural network is difficult in living mice. However, direct observation of cells and their behaviours both *in vitro* and *in vivo* have lately offered unique insight in the understanding of cellular interactions. Imaging the living brain has revealed unexpected details of the dynamics and the functional properties of neurons, astrocytes and microglia under conditions following stimulation or injury [25-27]. Nonetheless, limited work has been performed on imaging the living spinal cord due to technical difficulties. The close proximity of the animal’s heart and lungs to the spinal column can result in artefacts generated by the heartbeat and breathing movements which significantly impede the acquisition of steady images from the spinal cord of anesthetised mice [28]. As a result, imaging studies have been predominately performed on *ex vivo* spinal tissue [19,29]. Nevertheless, *in vivo* 2P-LSM was applied to simultaneously record central axons of projection neurons and microglia in the spinal cord [30,31].

In this present work, time-lapse and multiphoton microscopy as well as immunohistochemical and morphological studies were carried out to focus on the association of astrocyte-like cells with axons at early stages of their myelination. The differentiation of neural progenitor cells in our specific differentiation media resulted in the production of astrocytes and oligodendrocytes as previously reported [32,33]. In this media astrocyte lineage cells, as indicated by expression of GFAP, were the major cell type observed with 65% +/-1.5% of cells were immunoreactive for GFAP.

Previously, we had focused on oligodendrocyte-like cells and their interaction with axons in these cultures [19]; however, in this study we focus on astrocyte-like cells based on their morphology and immunohistochemistry of sister cultures. From the time-lapse sequences of myelinating cultures with exogenous GFP tagged cells it was observed that many of these flat labelled cells had a leading process projecting towards neurites. In the majority of cells, that leading process was highly dynamic, extending and retracting slowly during the time studied. Using fGFP tagged cells which labels membrane bound GFP spherical protrusions typical of blebs could be observed.

There are several reports showing an association between astrocytes and myelination in culture. The increase in GFAP-positive cells observed during development correlates with the normal developmental period of myelination in the spinal cord [34] and experimental studies in cell culture show that astrocytes induce oligodendrocytes to align their processes with axons [35]. Moreover, it has been proposed that reactive astrocytes are functionally linked to axon elongation, in a developed PLGA/F127 tube that was implanted in to the spinal cord for analysis of infiltrated cells and elongated spinal axons [36]. Blebs have been implicated in many cell functions including cell movement. We previously identified bleb formation while oligodendrocytes were interacting with neurites/axons [19] and in this study we see similar changes in the cell membrane for astrocytes.

In support of our findings, others have reported that astroglial processes play a key role in glial/axonal interactions. For example astrocyte processes contact neuronal somata and enwrap active synaptic terminals [37]. There has been some link to the formation of membrane protrusions and sorting of proteins, present in intracellular membrane compartments which later are transported via active protrusions and membrane waves to the corresponding specific plasma membrane domain. Spine-like astrocytic protrusions have been observed by electron microscopy into large axon terminals [38]. However, very little is known about the underlying mechanisms that initiate and control the formation and regulation of astrocyte protrusions. These studies have been carried out from static observations rather than dynamically over time. Moreover, it is known that the presence of astrocyte in oligodendrocyte/neurite cultures enhances myelination and regulate OPC function. For example, synthesis and release of leukemia inhibitory factor (LIF) from astrocytes is stimulated by action potential firing [23] which promotes myelination. Ciliary neurotrophic factor (CNTF) secreted by astrocytes has also been shown to be an important survival factor for oligodendrocytes [39] and promotes myelination [6]. Also, FGF2 an OPC mitogen [40] is predominantly expressed by astrocytes in response to myelin damage [41].

To confirm if similar changes were detected in the myelinating cultures *in vitro* occur *in vivo,* we injected neurospheres into the spinal cord of *shiverer* mice. Neurospheres can myelinate the non myelinated fibres in *shiverer* mice [17,18] and as expected transplanted cells were found distributed throughout all regions of the engraftment area. However, we found that colocalisation of GFAP and GFP immunoreactivity could be detected most intensely at the needle track. The neurospheres injected at this traumatic site differentiated into astrocytes and therefore it is possible to speculate the cellular environment of the needle tract secreted factors that promoted astrocytic differentiation. Exogenous GFP-MBP-positive cells were present both at the injection site and locations distant from it although astrocytes remained mainly at the injection site.

*Ex vivo* and *in vivo* multiphoton observations followed by immunohistochemical studies gave an insight into the morphology of the glial cells from the GFP-positive neurospheres after transplantation. Astrocyte had thick processes and were larger in size in general when compared to oligodendrocytes which exhibited multiple fine processes emanating symmetrically from a central cell body;as confirmed by GFAP and MBP staining respectively. Rapid structural changes of astroglial-like processes were seen from time-lapse laser scanning recordings of *ex vivo* spinal tissue after transplantation of GFP-labelled cells. Time-lapse imaging of the *ex vivo shiverer* spinal cord showed dynamic morphological changes in the distribution of the cytoplasm suggesting movement of cGFP-transplanted cell processes. Spontaneous transient extension of astroglial filopodia has been reported in brainstem slices of transgenic mice [37]. Although we were able to visualise a GFP positive transplanted cell in a live mouse, further work on data acquisition is required to remove breathing artifacts.

## Conclusion

We have shown that astrocytes are very dynamic cells forming membranous blebs when in close contact with both axons and oligodendrocytes. Future experiments blocking mechanisms of blebs and astroglial process propagation and arresting actin filament motility may provide evidence to further support the dynamic role of astrocytes prior to and during myelination.

## Methods

### Mice

The following mice were used and maintained in Glasgow University Veterinary Research Facility: homozygous *shiverer* mice (*shiv/shiv*) on the C3H/101 genetic background and mice expressing enhanced GFP under the *β-actin* promoter [42] on the C57BL/6 genetic background (C57BL/6-Tg(ACTB-EGFP)1Osb/J). All experimental mice were bred at the facility. Mice had access to food and water, *ad libitum*. Furthermore, all procedures were carried out in accordance with the guidelines, set forth by the Animals Scientific Procedures Act, under a project license (No. 6003895) granted by the UK Home Office and with the approval of the University of Glasgow Ethical Review Process Applications Panel.

### Isolation and culture of GFP-labelled and wild type neurospheres

Neurospheres were generated from striata of *β-actin* GFP-transgenic mice expressing cytoplasmic GFP (cGFP) or wild type mice as previously described [24], based on methods from Reynolds and Weiss [43,44]. Briefly, the striata were dissected from postnatal day 1 embryos and mechanically dissociated and triturated through a glass Pasteur pipette to produce a cell suspension. The dissociated cells were spun at 800 rpm and the cell pellet reconstituted in 20 ml of neurosphere medium, consisting of DMEM/F12 (1:1, DMEM containing 4,500 mg/L glucose), supplemented with 0.105% NaHCO_3,_ 2 mM glutamine, 5,000 IU/ml penicillin, 5 mg/ml streptomycin, 5.0 mM HEPES, 0.0001% bovine serum albumin, (all Invitrogen), 25 μg/ml insulin, 100 μg/ml apotransferrin, 60 μM putrescine, 20 nM progesterone, and 30 nM sodium selenite (all Sigma). The suspension was then placed into a 75 cm^3^ tissue culture flask (Greiner, UK) and supplemented with 20 ng/ml mouse submaxillary gland epidermal growth factor (EGF, Peprotech, UK). Every 2-3 days, 5 ml neurosphere medium and 4 μl EGF (20 ng/ml) were added into the flask [44] and the cultures maintained at 37°C, in a humidified atmosphere of 7% CO_2_/93% air. GFP neurospheres were used in both the *in vitro* and *in vivo* experiments. In some experiments the neurospheres were labelled with farnesylated GFP (fGFP) by lentivral transduction (lentivirus gift from Prof J Verhaagen, Netherlands Institute for Neuroscience, NIN) which remained bound to the plasma membrane as previously described [19]. Briefly triturated neurospheres were incubated with 10 μl of 2.2 × 10^9^ transducing units (TU)/ml of the viral supernatant overnight and maintained in neurosphere medium.

Myelinating cultures were generated from E13.5 (embryonic day) pups as described previously [24,45,46]. Briefly the spinal cord was dissected, the meninges removed, and minced using a scalpel blade prior to enzymatic dissociation (100 μl of 2.5% trypsin, Invitrogen, Paisley, UK and 100 μl of 1.33% collagenase, ICN Pharmaceuticals, UK). Enzymatic activity was stopped by the addition of 1 ml of a solution (SD) containing 0.52 mg/ml soyabean trypsin inhibitor, 3.0 mg/ml bovine serum albumin and 0.04 mg/ml DNase (Sigma, UK) to prevent cell clumping. The cells were triturated through a glass pipette and spun at 800 rpm and the pellet resuspended in 5 ml of plating medium [PM: 50% low glucose DMEM, 25% horse serum, 25% HBSS (Hanks balanced salt solution without Ca^2+^ and Mg^2+^) and 2 mM L-glutamine (Invitrogen). The dissociated mixed spinal cord cells were plated onto PLL-coated coverslips at a density of 150,000 cells/100 μl and left to attach for 2 hr in the incubator, after which 300 μl of PM and 500 μl of differentiation medium (DM) was added, which contained DMEM 4,500 mg/mL glucose, 10 ng/ml biotin, 0.5% hormone mixture (1 mg/mL apotransferrin, 20 mM putrescine, 4 μM progesterone, and 6 μM selenium) (formulation based on N2 mix [47]), 50 nM hydrocortisone, and 0.5 μg/ml insulin (all reagents from Sigma) was added. Cultures were maintained by replacing half of the medium with fresh medium three times a week. After 12 days in culture, insulin was excluded from the DM. The cultures were maintained for up to 28 days, in a humidified atmosphere of 7% CO_2_/93% air, at 37°C. For time-lapse imaging in some cases, ascorbic acid (0.5 μl/1 ml) was added to the medium to enhance cell survival.

### Laminectomy and transplantation of neurospheres *in vivo*

The method of Edgar et al., 2004 was followed [17]. Briefly a short vertical incision was made with a scalpel blade over the thoraco-lumbar region of the spine, from anterior to posterior. With the aid of an operating microscope, the transverse laminae of a vertebra were broken (laminectomy) to reveal the spinal cord. A small opening was made in the dura with a sterile needle and a cell suspension of cGFP or fGFP neurospheres which had been actively growing in neurospheres medium containing EGF, was injected using a CellTram Oil manual micromanipulator (Eppendorf Ltd, Cambridge, UK) at a rate of 1 μl/min, using a glass microelectrode that was inserted into the exposed spinal cord. One injection was made into the dorsal spinal cord, avoiding the midline dorsal vein. A total volume of 3-5 μl of cell suspension containing, approximately 5 × 10^4^ cells/μl was injected, at the depth of < 1 mm. The microelectrode was left in place for an additional 2 min to minimise back-flow of cells. No immunosuppressant treatment was used. Mice were sacrificed at various time points 3 days, 7-10 days, 14 days and 4 weeks (±3 days) after transplantation.

### Tissue processing and immunocytochemistry

Animals were euthanized with CO_2_ and perfused transcardially with 10 ml of saline, followed by 20 ml of 4% paraformaldehyde. The spinal cords were dissected and placed in 0.1 M glycine in PBS, then cryoprotected in 20% sucrose overnight at 4°C followed by embedding in OCT and rapid freezing in liquid nitrogen cooled-isopentane. The rostro-caudal segment at the lumbar thoracic junction of the spinal cord encompassing the transplant site was embedded and cryosectioned dorso-ventrally. Cryostat serial sections (10 μm) were cut, mounted onto APES-coated slides and stored at -20°C. Before immunostaining, sections were air dried at room temperature (RT) for 10-20 min and then rehydrated in PBS for 10 min. The sections were permeabilised with methanol at -20°C for 10 min and blocked with 10% normal goat serum in PBS for 1 hour at RT followed by incubation in primary antibody in the same blocking solution overnight at 4°C. Antibodies used were: rabbit anti-GFP (1:1000, Abcam), mouse anti-GFP (1:250, Abcam) for GFP transplanted cells, rat anti-MBP (1:500, Serotec) for mature oligodendrocytes, the O4 antibody for oligodendrocytes [48]; 1:1 hybridoma, IgM), mouse anti-GFAP (1:1000, Sigma) for astrocytes and mouse anti-phosphorylated neurofilament SMI-31 for neurites. The slides were washed three times with PBS and then incubated with appropriate fluorescent-conjugated secondary antibodies (Cambridge Biosciences) for 1 hour at RT. The slides were washed three times with PBS and then mounted in Citifluor antifade (Citifluor Ltd, UK) mounting medium.

### Quantification of transplanted cells in the *shiverer* mouse

10x 10 micron thick sections were collected over the transplant site for each experiment and immuolabelled with cell type markers. Using Image J and a macro (generated in the lab) that created homocentric circles 150 microns apart, allocated from the epicentre of the injection area, the distance of oligodendroglial cells (MBP), astrocytes (GFAP) and other cells (GFAP/MBP–ve) from the transplantation site was measured. Any cells found within the borders of two circles were counted and plotted against distance from the injection site.

### Maintenance of the explants for *ex vivo* imaging

The protocol was based on studies by Kerschensteiner and colleagues [49]. All procedures were performed in a combination of F12 + L-glutamine, CO_2_ independent medium and DMEM + Glutamax (4 g/L D-glucose) or Neurobasal A medium (Invitrogen) that had been bubbled with 95% O_2_ and 5% CO_2_ for at least 15 min before imaging. During dissection, the mouse was placed on aluminium foil with ice underneath to protect the tissue from hypoxia. During imaging the explant was superfused with pre-warmed O_2_-bubbled medium. The temperature of the explant was maintained at 35-37°C using a heating stage, superfused with pre-warmed medium.

### Multiphoton microscopy

Multiphoton microscopy was performed using a LaVision BioTec 2-photon TRIM scope, and a Zeiss 7MP. The LaVision system consisted of a Nikon Eclipse TE2000 inverted stand, Olympus long working distance 20x 0.95 NA water immersion objective and Coherent Chameleon II laser tuned to 830 nm. Fluorescence was detected using non-descanned detectors (NDD, Hamamatsu H6780-01-LV 1 M for < 500 nm detection and H6780-20-LV 1 M for > 500 nm detection). A dichroic filter (Chroma 475 DCXR) was used to separate spectrally the second harmonic signal, when present, from the GFP emission of the transplanted cells. Band pass filters (Semrock 435/40 and Chroma 525/50) were used to further filter the emission for the SHG and GFP channels respectively. The Zeiss system consisted of an Axio Imager upright stand, 20x, 1.0 NA water immersion objective, and Chameleon II laser. To keep tissue stationary during *ex vivo* imaging it was glued, dorsal part side up to a plastic cover slip with cyanoacrylate (Vetbond, 3M Health Care Ltd, Leicestershire, UK). The cover slip was cut to fit in a perfusion chamber (Harvard Apparatus ltd, Kent, UK) where it was held in place by grease. The perfusate was equilibrated with oxygen and thermo-stated to 35-37°C. The chamber was placed on the motorized stage of the upright microscope and fluorescent cells located using epifluorescence with (blue) excitation. The wavelength of the laser was set to either 840 nm (to excite predominantly CFP) or 940 nm (to excite predominantly GFP). Detection channels selected light with wavelength < 485 nm (for CFP) and 500 - 550 nm (for GFP). For *in vivo* imaging a laminectomy was performed and the mouse placed dorsally in a custom made V-shaped plinth with adjustable legs and a small opening to allow the microscope lens (inverted) to access the spinal cord. Repetitive administration of anaesthetic drugs every 2 hours, kept the heart beat and breathing relatively low to facilitate the acquisition of images during time-lapse.

### Time-lapse microscopy

The Nikon time-lapse microscope TE2000 is fitted with a Nikon perfect focus system (PFS) to maintain focus over the imaging. The PSF only works with glass bottom dishes. So 35 mm glass bottom microwell Petri dishes, with 14 mm diameter of microwell (MakTek Corporation, MA, USA) were used for all the time-lapse experiments. The system has a temperature-controlled 37°C chamber, provided with an oxygen supply and images were acquired using a 40x short distance 0.75 NA air objective. Analysis was performed with MetaMorph (Version 5) imaging software, which compensate for stage shift, vibration or similar small whole field movement that can occur during time-lapse acquisition.

## Competing interests

The authors have no commercial or financial association that might create a competing interest in connection with the submitted manuscript.

## Authors’ contributions

Conceived and designed the experiments: KI SCB JME Performed the experiments: KI JME DS. Analyzed the data: KI SCB JME. Contributed reagents/materials/analysis tools: KA. Wrote the paper: KI SCB JME. All authors read and approved the final manuscript.

## Supplementary Material

Additional file 1 **Time-lapse sequence over 21 hours of a dynamic fGFP labelled process from flat astrocyte-like cells in association with a neurite/axon- like structure.** A flat fGFP tagged cell process can be seen to dynamically move over a neurite and form a large membranous bleb-like structure of around 10μm in diameter. Additional membranous blebs can be seen to form over time indicating an active motile, membrane. Click here for file

Additional file 2 **Time-lapse imaging of membrane protrusions of fGFP-positive cells in *shiverer *** **myelinating cultures after 21 DIV.** Time-lapse imaging was carried out for 24 hours with 3 min intervals. Membrane protrusions could be seen to form which changed shape and appeared to be eventually deposited on the axonal surface. Click here for file

Additional file 3 ***Ex vivo *** **time-lapse of cGFP-expressing cells typical of astrocytes revealed their dynamic behaviour.** cGFP expressing neurospheres were transplanted into the cord 12 days previously. The non-fixed cord was removed and visualised as *ex vivo* tissue by time lapse over a short time frame. A cGFP tagged astrocyte-like cell could be seen to change the pattern of GFP localisation which suggested a dynamic spontaneous extension of a cell process. Click here for file

Additional file 4 ***In vivo*** **cGFP-labelled neurospheres were transplanted and a cGFP-labelled cell with long processes resembling an astrocyte was visualised *in vivo *** **over a short time frame using time-lapse.** Although the movement of breathing could be seen the GFP tagged transplanted cell was clearly visible. Click here for file
